# An Acute Exposure to Muscle Vibration Decreases Knee Extensors Force Production and Modulates Associated Central Nervous System Excitability

**DOI:** 10.3389/fnhum.2017.00519

**Published:** 2017-10-25

**Authors:** Robin Souron, Thibault Besson, Chris J. McNeil, Thomas Lapole, Guillaume Y. Millet

**Affiliations:** ^1^Human Performance Laboratory, Faculty of Kinesiology, University of Calgary, Calgary, AB, Canada; ^2^Laboratoire Interuniversitaire de Biologie de la Motricité, UJM Saint-Etienne, University Lyon, Saint-Etienne, France; ^3^School of Health and Exercise Sciences, University of British Columbia, Kelowna, BC, Canada

**Keywords:** cortical voluntary activation, corticospinal excitability and inhibition, local vibration, thoracic electrical stimulation, transcranial magnetic stimulation

## Abstract

Local vibration (LV) has been recently validated as an efficient training method to improve muscle strength. Understanding the acute effects may help elucidate the mechanism(s). This study aimed to investigate the effects of a single bout of prolonged LV on knee extensor force production and corticospinal responsiveness of vastus lateralis (VL) and rectus femoris (RF) muscles in healthy young and old adults. Across two visits, 23 adult subjects (20–75 years old) performed pre- and post-test measurements, separated by 30-min of either rest (control; CON) or LV. Maximal voluntary contraction (MVC) force was assessed and transcranial magnetic stimulation (TMS) was used to evaluate cortical voluntary activation (VA_TMS_) as well as the motor evoked potential (MEP) and silent period (SP). In 11 young adults, thoracic electrical stimulation was used to assess the thoracic motor evoked potential (TMEP). Although MVC decreased after both CON (−6.3 ± 4.4%, *p* = 0.01) and LV (−12.9 ± 7.7%, *p* < 0.001), the MVC loss was greater after LV (*p* = 0.001). Normalized maximal electromyographic (EMG) activity decreased after LV for both VL (−25.1 ± 10.7%) and RF (−20.9 ± 16.5%; *p* < 0.001), while it was unchanged after CON (*p* = 0.32). For RF, the TMEP and MEP/TMEP ratio decreased (*p* = 0.01) and increased (*p* = 0.01) after LV, respectively. Both measures were unchanged for VL (*p* = 0.27 and *p* = 0.15, respectively). No changes were reported for TMS-related parameters. These results confirm our hypothesis that modulations within the central nervous system would accompany the significant reduction of maximal voluntary force. A reduced motoneuron excitability seems to explain the decreased MVC after prolonged LV, as suggested by reductions in maximal EMG (all subjects) and TMEP area (data from 11 young subjects). A concomitant increased cortical excitability seems to compensate for lower excitability at the spinal level.

## Introduction

In recent years, a prolonged (20–60 min) period of local vibration (LV) has been validated as an efficient training method to increase muscle strength in healthy young subjects for plantar flexor (Lapole and Pérot, 2010) and dorsiflexor muscles (Souron et al., 2017) as well as for knee extensor muscles in old subjects (Tankisheva et al., 2015). Because this method does not require an active contribution and may be applied while the subject is relaxed (sitting, lying down), prolonged LV may be particularly beneficial for specific populations; e.g., older subjects (Tankisheva et al., 2015). In order to better consider chronic uses, it is important to understand acute effects. A single exposure to prolonged LV can induce a significant neuromuscular workload in healthy young subjects, as indicated by the decrease of maximal voluntary contraction (MVC) force reported after 20–30 min of LV for knee extensor (Kouzaki et al., 2000; Jackson and Turner, 2003), plantar flexor (Ushiyama et al., 2005) and first dorsal interosseous muscles (Shinohara et al., 2005).

As with neuromuscular fatigue induced by “traditional” exercise, the vibration-induced decreased MVC may arise from central and/or peripheral mechanisms. An acute exposure to 20–30 min of LV failed to modulate mechanisms downstream of the neuromuscular junction (Ushiyama et al., 2005; Herda et al., 2009), so it appears the decreased MVC is explained by neural alterations, likely induced by the well-reported strong activation of Ia afferents by vibratory stimuli (Burke, 1980). When vibration exposure is prolonged, a decrease in alpha motoneuron activity has been reported (Bongiovanni and Hagbarth, 1990; Bongiovanni et al., 1990). This is likely linked to an attenuation of Ia afferent discharge, supported by reductions in spinal loop excitability after prolonged LV (Heckman et al., 1984; Hayward et al., 1986; Fry and Folland, 2014; Farabet et al., 2016). Based on the lack of changes in the F-wave (Christova et al., 2011; Lapole et al., 2012b), it has been argued that intrinsic motoneuronal excitability is not impacted after prolonged LV; however, many authors have suggested the F-wave provides a flawed measure of motoneuron excitability (for review, see McNeil et al., 2013). Responses to electrical stimulation of the descending corticospinal tract at the level of the mastoids (cervicomedullary motor evoked potential, CMEP) or thoracic spine (thoracic motor evoked potential, TMEP) provide the most direct assessment of the motoneuron pool’s responsiveness to synaptic input because: (i) a large proportion of the response is monosynaptic for the upper limb (Petersen et al., 2002) and probably the lower limb (Martin et al., 2008); and (ii) evidence exists that descending tracts are not influenced by presynaptic inhibition (Nielsen and Petersen, 1994). Despite the utility of corticospinal tract stimulation, to date, no study has examined the response of CMEPs or TMEPs to prolonged LV.

Although it is intuitive that prolonged LV has the potential to modulate the spinal level, transient vibration increases Ia afferent input to both motor and sensorimotor cortices as well as the contralateral supplementary motor area (Naito and Ehrsson, 2001; Romaiguère et al., 2003) suggesting prolonged LV could also have a supraspinal impact (Pamukoff et al., 2016a,b). The motor evoked potential (MEP) in response to transcranial magnetic stimulation (TMS) of the motor cortex can provide an index of cortical excitability following an intervention, if one considers the MEP along with a valid measure of motoneuronal excitability (i.e., normalizes the MEP to the CMEP or TMEP). It is unclear how prolonged LV affects cortical excitability because no previous study has normalized the MEP in this manner. Furthermore, the data which exist are equivocal as MEPs have been reported to increase (Steyvers et al., 2003; Lapole et al., 2012b), decrease (Steyvers et al., 2003; Farabet et al., 2016), or remain unchanged (Lapole et al., 2012b; Lapole and Tindel, 2015) after prolonged LV using frequencies from 50 Hz to 100 Hz and amplitudes from 0.2 mm to 1 mm.

When applied during a voluntary contraction, TMS may also inform about GABA_B_-mediated intracortical inhibition through the assessment of the silent period (SP), which is the interval of muted electromyographic (EMG) activity that follows the MEP (Terao et al., 2000). More evidence is required, but we recently showed that 30 min of LV (frequency: 100 Hz; amplitude: 1 mm) did not impact SP duration in the tibialis anterior (Farabet et al., 2016). Finally, TMS may also assess the level of corticospinal drive (voluntary activation, VA_TMS_) by evoking superimposed twitches (SIT) during voluntary contraction (Todd et al., 2003). Our team recently showed no changes in VA_TMS_ for dorsiflexor muscles after a 30-min LV period (Farabet et al., 2016) but the effects of LV on VA_TMS_ still remain to be tested on knee extensor muscles, an important functional muscle group (Hurley et al., 1998) which is susceptible to a fatigue-related impairment of voluntary activation (Millet et al., 2011; Jubeau et al., 2014).

While chronic use of LV has been reported to be an efficient means to increase muscle strength (Lapole and Pérot, 2010; Lapole et al., 2013; Souron et al., 2017), a greater understanding of acute effects of LV is needed to inform effective long-term use (training). Thus, this study investigated the effects of a single exposure to prolonged LV on force production capacities and associated corticospinal properties (e.g., VA_TMS_, TMEPs, MEPs, SPs) of knee extensor muscles in healthy adults. Due to the potential neuromuscular benefits LV could have in a less mobile population (e.g., elderly), we included a small sample of old subjects to probe the possibility of age-related differences in the response to prolonged LV. Without consideration of age, we hypothesized that modulations in corticospinal excitability would accompany the significant reduction of MVC force.

## Materials and Methods

### Subjects

Twenty-three healthy subjects volunteered for this study after giving their informed consent. The study included 15 young (11 men and 4 women, age: 28 ± 6 year; height: 175 ± 8 cm; weight: 70 ± 9 kg) and eight old (2 men and 6 women, age: 65 ± 5 year; height: 168 ± 15 cm; weight: 72 ± 10 kg) subjects. This study was carried out in accordance with the recommendations of the local ethics committee (Conjoint Health Research Ethics Board, University of Calgary) with written informed consent from all subjects. All subjects gave written informed consent in accordance with the Declaration of Helsinki. The protocol was approved by the local ethics committee (Ethics ID: REB #15-2752). All subjects were free of lower-limb injury during the previous 3 months, had no contraindications to TMS (Rossi et al., 2011), and no acute or chronic neurological disorders and trauma. Subjects were not informed of the investigators’ hypotheses, only that the effect of LV was being tested. They were instructed to abstain from caffeine for a minimum of 12 h before each session.

### Experimental Design

Subjects completed a familiarization session to get used to the correct development of knee extension maximal isometric force and the electrical and magnetic stimulations. Then, subjects were tested twice in random conditions (crossover design) on two separate days with a 4-week interval. No specific recommendations were given to the subjects during the 4-week period. They were only asked not to change their usual physical activities. The subjects were randomly divided into two groups with the first one starting with the control condition (CON) and the second one starting with the vibration condition (LV). This was inverted for the second session. Both sessions took place at the same time of the day, to avoid circadian variations.

### Subject Set-Up

Knee extensor force of the right lower limb was measured during voluntary contraction by a calibrated force transducer (Omega Engineering Inc., Stamford, CT, USA). Subjects were seated upright in a chair with both right knee and hips at 90° of flexion. Movement of the upper body was minimized using three belts across the thorax and waist. During all measurements, subjects were provided real-time feedback of the force trace on a screen, and guidelines were added when subjects had to maintain a targeted submaximal level of MVC.

**Figure 1:**
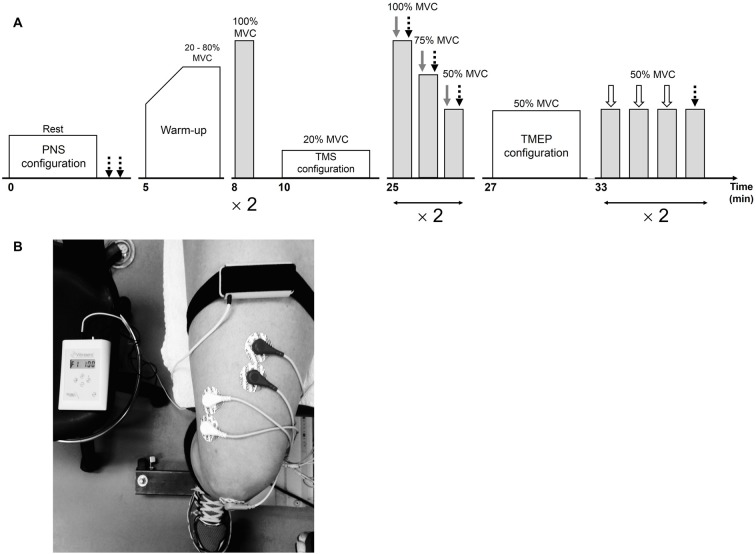
Overview of the neuromuscular testing protocol prior to the interventions **(A)**. Following the interventions, the warm-up and stimulation configurations were not completed. Peripheral nerve stimulation (PNS), transcranial magnetic stimulation (TMS) and electrical stimulation over the thoracic spine are represented by dotted black, gray and white arrows, respectively. The position of the vibratory device on the leg during the 30-min vibration period is illustrated in **(B)**.

EMG activity of the vastus lateralis (VL), rectus femoris (RF) and biceps femoris (BF) was recorded with pairs of self-adhesive surface electrodes (Meditrace 100, Covidien, Mansfield, MA, USA) placed at the distal end of each muscle of interest in a bipolar configuration with a 30-mm interelectrode distance (Figure 1B). The reference was placed on the patella. Low impedance (<5 kΩ) was obtained by shaving, gently abrading the skin and then cleaning it with isopropyl alcohol. EMG signals were amplified with an octal bio-amplifier (ML138, ADInstruments, Bella Vista, Australia), bandpass filtered (5–500 Hz) and analog-to-digitally converted at a sampling rate of 2000 Hz by PowerLab System (16/35, ADInstruments). All data were analyzed offline using Labchart 8 software (ADInstruments).

### Local Vibration

The vibrating device (VB 115, Techno Concept, Mane, France) was applied locally and strapped directly on the right RF muscle (40% of the muscle length from the upper edge of the patella to the anterior superior iliac spine) using elastic Velcro fasteners (Figure 1B) for the LV condition. During CON, the vibrating device was also placed on the leg but was turned off. The right leg was the dominant one for 21 out of 23 subjects (the two subjects with the left leg as the dominant one belonged to the young group). As Ia afferents are sensitive to a small vibration amplitude (Roll et al., 1989) and fire synchronously with vibration frequencies up to 80–120 Hz (Roll and Vedel, 1982; Roll et al., 1989), vibration characteristics were 100-Hz frequency and 1-mm amplitude in the present study, as already set in previous works led by our team (Farabet et al., 2016).

### Peripheral Nerve Stimulation

Single rectangular pulses with 1-ms duration and 400 V maximal output voltage were delivered via constant-current stimulator (DS7A, Digitimer, Hertfordshire, UK) to the right femoral nerve via a 30-mm-diameter surface cathode (Meditrace 100) taped to the skin into the femoral triangle and a 50 × 90 mm anode (Durastick Plus; DJO Global, Vista, CA, USA) in the gluteal fold. To determine the optimal intensity of stimulation for M-wave measurement, single stimuli were delivered incrementally by steps of 10 mA until resting M-wave (M_max_) and twitch amplitudes plateaued. The optimal intensity was then increased by 30% to confirm supramaximality.

### Transcranial Magnetic Stimulation

The left motor cortex was stimulated by a magnetic stimulator (Magstim 200^2^, The Magstim Company Ltd., Whitland, UK) with a 110-mm double-cone-coil (maximum output of 1.4 T). The coil was positioned to induce a postero-anterior current and manually controlled by the same investigator throughout all the testing sessions. A cervical collar was worn during all TMS measures to stabilize the head and neck and a swim cap was worn to ensure consistent coil placement relative to the optimal position. To determine this site, six marks were drawn on the cap: the vertex, 1 and 2 cm posterior to the vertex and 1 cm to the left of these three marks along the midline. Optimal coil position was determined as the site eliciting the largest SIT as well as VL and RF MEP amplitudes, with a small MEP amplitude in the antagonist BF, in response to stimulation at a known suprathreshold stimulator output (50% maximum) during a 10% MVC knee extension. When optimal agonist MEPs and peak force were not recorded at the same coil position, the optimal coil position was chosen according to MEP amplitude, because the evoked peak force may be influenced by activation of other muscles (Todd et al., 2016). This position was marked on the swim cap and was the same for both RF and VL muscles.

**Figure 2:**
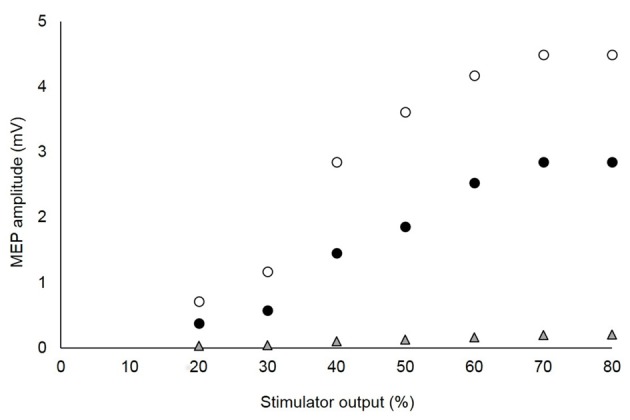
Stimulus-response curves of motor evoked potentials (MEPs) recorded from agonist and antagonist muscles in a single, representative subject. The optimal stimulation intensity was that which showed a plateau of the vastus lateralis (VL; white circles) and rectus femoris (RF; black circles) MEPs, without a marked increase of the MEP for the antagonist biceps femoris (BF; gray triangles). For this subject, 70% of the maximal stimulator output was chosen as the optimal intensity.

As per our recent recommendation (Temesi et al., 2014), the optimal stimulus intensity was determined from SIT and MEP stimulus-response curves obtained during brief (2–3 s) voluntary contractions at 20% MVC. In brief, intensities of 20, 30, 40, 50, 60, 70 and 80% stimulator output were tested in random order. At each intensity, four contractions were performed at 10 s intervals. A rest period of 10 s separate each intensity. In one subject, the SIT and MEP amplitudes did not plateau using these intensities so stimulator outputs of 90 and 100% were added to reach a plateau. The optimal stimulation intensity was considered the lowest which showed a plateau for VL and RF MEPs as well as the SIT. The MEP evoked in the antagonist BF was also measured to make sure that its size was low compared to those recorded in VL and RF and did not increase substantially with an increase in stimulator output (Figure 2). Mean TMS intensities were 67 ± 12% and 65 ± 11% stimulator output for CON and LV, respectively.

### Thoracic Electrical Stimulation

To find the optimal intensity used to elicit TMEPs, the descending corticospinal tract was stimulated over the thoracic level during brief voluntary knee extensors contractions at 50% MVC. Responses were collected during contraction because large TMEPs are difficult to acquire at rest, when motoneuron excitability is low (Martin et al., 2008). A constant-current stimulator (DS7A, Digitimer) was used to evoke TMEPs by passing a single rectangular electrical pulse with 1-ms duration and 400 V maximal output voltage between surface electrodes positioned between the spinous processes of T3 and T4 (cathode) and 5–10 cm above (anode; Ugawa et al., 1991; Aboodarda et al., 2015). The intensity of stimulation was incrementally increased by steps of 5 mA until the TMEP peak-to-peak amplitude was approximately 20%–30% of the M-wave obtained at 50% MVC (M_max_50_, see below). Because of: (i) the discomfort produced by the electrical stimulus of the thoracic spine; and (ii) the difficulty to obtain responses of sufficient size (McNeil et al., 2013), the old subjects were not asked to perform TMEP measurements. Thus, only 11 young subjects (9 men and 2 women) underwent electrical stimuli of the thoracic spine (4 out of the 15 young subjects did not performed TMEP measurements due to the discomfort produced by thoracic electrical stimulation). The mean thoracic electrical stimulation intensity was 53.0 ± 14.8 mA and 52.5 ± 16.4 mA for CON and LV, respectively.

### Experimental Procedures

The experimental procedures are illustrated in Figure 1. A testing session consisted of neuromuscular measurements performed before (PRE; Figure 1A) and after (POST) a 30-min resting (CON) or LV period (LV). First, the optimal position and intensity of stimulation for peripheral nerve stimulation (PNS) were determined in order to assess resting M-waves (M_max_) for RF and VL. Then, after a standardized warm-up consisting of three brief (~3 s) submaximal knee extensor isometric voluntary contractions at 20, 40, 60 and 80% MVC, the subjects performed two brief (~3 s) MVCs interspaced by 1 min. Optimal coil position and intensity for TMS were then established. Cortical voluntary activation, i.e., VA_TMS_, was calculated using two series of three brief (~5 s) contractions (100, 75 and 50% MVC), with single TMS and PNS pulses delivered during each contraction. Rest periods of 15 s and 1 min were provided between contractions within a series and between the series, respectively. To permit accurate determination of the SP, subjects were instructed to momentarily (<1 s) contract as strong and quickly as possible immediately after delivery of the single TMS pulse. Finally, optimal position and intensity for electrical stimulation over the thoracic spine was determined and two series of four brief (~3 s) contractions at 50% MVC were performed. Rest periods of 15 s and 1 min were provided between contractions within a series and between the series, respectively. Thoracic stimulation (first three contractions) or PNS (last contraction) was applied to elicit TMEPs and M-waves, respectively. The subjects who opted out of thoracic stimulation still performed these four submaximal voluntary contractions at 50% MVC without receiving any form of stimulation. Stimulation sites and intensities were not re-established after the CON or LV period so subjects started with the first series of VA_TMS_ measurements 10 s after the intervention (the second series began at ~1:55). The two series of contractions at 50% MVC (to collect TMEP and M_max_) started at ~3:40 and ~5:35. Considering the time-course of the post-LV assessments, it is certainly possible there was an attenuation of some effects during our measurement period, representing a limitation in the interpretation of our normalized evoked EMG data.

### Data Analysis

**Figure 3:**
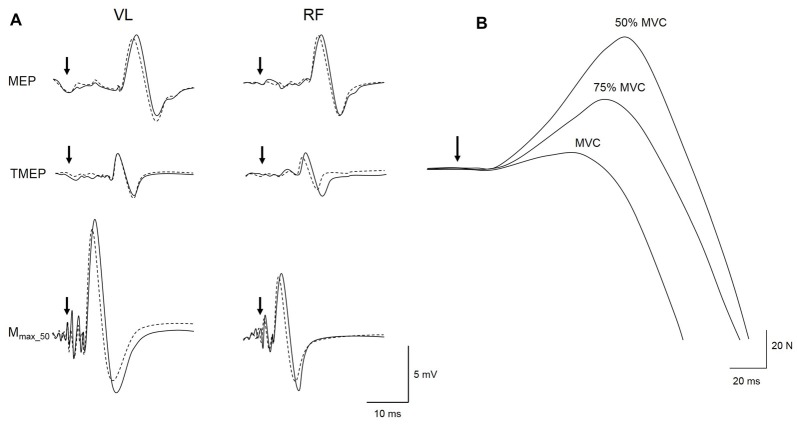
Representative trace of a MEP, thoracic motor evoked potential (TMEP) and M-wave (M_max_50_) recorded for local vibration (LV) session at PRE (solid line) and POST (dotted line) during a 50% maximal voluntary contraction (MVC) submaximal voluntary contraction on VL and RF muscles **(A)**. Raw traces from a single subject of the TMS superimposed twitches (SIT) evoked during maximal (MVC) and submaximal voluntary contractions at 75 and 50% MVC are displayed in **(B)**. Black arrows represent the time of magnetic or electrical stimulations.

As results for M-wave, MEP and TMEP peak-to-peak amplitudes and areas behaved similarly, only areas are reported. M-wave, MEP and TMEP areas were measured between cursors placed at the beginning, i.e., initial deflection from baseline, and the end, i.e., second horizontal crossing, of the evoked potentials (see Figure 1D in Martin et al., 2006). Evoked EMG data (M-wave, MEP, TMEP and SP) are presented as the average values of all responses collected pre- and post-intervention. A representative trace of the three types of evoked potentials obtained during a submaximal voluntary contraction at 50% MVC for PRE and POST sessions in LV is displayed in Figure 3.

#### PNS

M-wave areas were measured from electrical nerve stimulations in both relaxed (M_max_) and contracted VL and RF (M_max_50_ and M_max_MVC_ elicited during 50% and 100% MVC, respectively). In parallel to M_max_ measurement, the amplitude of the peak twitch (Pt) evoked during a relaxed state (Figure 1A) was also determined as a mean of the recorded twitches.

#### Force and Voluntary EMG

Maximal force was considered as the peak value recorded before the TMS stimulus from the four MVCs pre- and the two MVCs post-intervention. Root mean square (RMS) EMG was calculated for VL (RMS_VL_) and RF (RMS_RF_) muscles over a 500-ms period after maximal force. RMS_VL_ and RMS_RF_ were then normalized to M_max_MVC_.

#### TMS

VA_TMS_ was measured by the modified twitch interpolation technique (Todd et al., 2003). In brief, the estimated resting twitch (ERT) was determined as the y-intercept of a linear regression of SIT amplitude and absolute voluntary force during the two series of contractions at 100, 75 and 50% MVC (Figure 3B), i.e., one ERT derived from six data points. The regression of voluntary torque and the SIT torque was linear (i.e., *r* > 0.9; Hunter et al., 2006) for all series (0.91 < *r* < 0.99) so no data were excluded from VA_TMS_ statistical analysis. VA_TMS_ was then assessed with the equation:

(1) $${\text{VA}}_{\text{TMS}}=\left(\text{1}-{\text{SIT}}_{\text{MVC}}/\text{ERT}\right)\times \text{1}00$$

where SIT_MVC_ is the mean SIT evoked during the two contractions at 100% MVC.

The area of VL and RF MEPs elicited by TMS during 100, 75 and 50% MVC contractions were normalized to the M-wave measured at the same contraction intensity. As a maximal M-wave was not recorded from BF in the experimental protocol, MEP_BF_ are presented as absolute areas rather than a percentage of the maximal output of the motoneuron pool. The inability to standardize the relative activation of antagonist motoneurons may compromise the validity of the VA_TMS_ measured for the knee extensors (Todd et al., 2016). SP durations for VL and RF were determined visually and defined as the duration from the TMS stimulus to the return of continuous voluntary EMG, i.e., when clear EMG bursts could be identified (Taylor et al., 1996).

#### TMEP

VL and RF TMEP areas were normalized to M_max_50_ to account for possible post-intervention changes in peripheral excitability.

### Statistical Analysis

The sample size was calculated at the outset for an expected “medium” effect size (*f*^2^ = 0.25) for MVC change between conditions, with α level of 0.05, power (1–β) of 0.80 and correlation among repeated measures of 0.85. Thus, including at least 20 subjects was necessary to reach the desired power of 0.80 on a two-way repeated-measures ANOVA, i.e., condition (CON or LV) × time (PRE or POST). However, the sample size calculation was performed without taking the factor “age” into consideration. Therefore, 20 subjects would theoretically be enough for detecting time and condition effects, but not group effects. Thus, it is possible that age effects were not detected due to insufficient statistical power, representing one of the main limitation of the current study. Statistical analyses were performed with Statistica software (StatSoft Inc., Tulsa, OK, USA). All variables were normally distributed (Shapiro-Wilk normality test). For ANOVA analyses, homogeneity of variance was verified by Levene’s test. Three-way repeated measures ANOVA were performed for MVC and VA_TMS_ [group (young or old) × condition (CON or LV) × time (PRE, POST)], as well as for TMEP analysis [condition (CON or LV) × time (PRE, POST) × muscle (VL, RF)]. Four-way repeated measures ANOVA were performed for the other neuromuscular parameters, i.e., MEPs, SPs, EMG RMS and M-waves [group (young or old) × condition (CON or LV) × time (PRE, POST) × muscle (VL, RF)]. *Post hoc* analyses were performed using Newman-Keuls testing when the ANOVA identified significant differences. Partial eta square (pη^2^) was reported as an estimate of effect size, with pη^2^ ≥ 0.07 and pη^2^ ≥ 0.14 used as moderate and large effects, respectively (Cohen, 1988). We also calculated difference scores for each session and report their mean and 95% confidence interval. Statistical significance was set at *p* < 0.05. All data are presented in the text and figures as mean ± standard deviation (SD).

## Results

*P* values from all ANOVA analyses are presented in Table 1. No differences between the two conditions (i.e., CON or LV) were found at baseline (i.e., PRE) for any parameters (*p* > 0.05). Although insufficient statistical power could have prevented detection of age-related differences (i.e., caused a type II error), because there was no effect of the group (young vs. old) on the magnitude of changes reported in this study, as demonstrated by the lack of significant group × condition × time interaction, data from young and old participants were pooled.

**Table 1:** *p* values from all ANOVA analyses.

	Condition	Time	Group	Condition × time	Time ×group	Condition × time × group	Muscle	Condition × muscle	Muscle × group	Condition × muscle ×group	Muscle × time × group	Condition × muscle × time	Condition × muscle × time group
MVC	0.11	<0.001	0.002	0.03	0.04	0.70							
RMS	0.08	<0.001	0.003	<0.001	0.16	0.36	<0.001	0.37	0.002	0.85	0.80	0.98	0.25
VA_TMS_	0.67	0.02	0.06	0.30	0.26	0.52							
MEP_MVC_	0.80	0.75	0.30	0.93	0.27	0.12	<0.001	0.99	0.01	0.19	0.31	0.90	0.87
MEP_75_	0.68	0.75	0.10	0.24	0.23	0.91	<0.001	0.43	0.01	0.98	0.21	0.31	0.75
MEP_50_	0.22	0.45	0.08	0.20	0.89	0.42	<0.001	0.07	0.03	0.81	0.16	0.12	0.49
SP_MVC_	0.65	0.05	0.54	0.45	0.46	0.87	0.73	0.46	0.82	0.78	0.06	0.12	0.22
SP_75_	0.74	0.01	0.64	0.73	0.55	0.86	0.42	0.92	0.44	0.31	0.15	0.02	0.64
SP_50_	0.53	0.05	0.65	0.22	0.85	0.39	0.15	0.66	0.99	0.50	0.75	0.69	0.71
TMEP	0.52	0.15		0.12			0.003	0.96				0.002	
MEP/TMEP	0.93	0.14		0.08			0.27	0.07				0.01	
M_max_	0.76	0.52	<0.001	0.94	0.13	0.77	<0.001	0.61	0.05	0.79	0.53	0.43	0.31
M_max_50_	0.61	0.01	0.001	0.84	0.001	0.57	<0.001	0.46	0.12	0.82	0.72	0.70	0.41
Pt	0.27	<0.001	0.03	0.88	0.03	0.98							

*MVC, maximal voluntary contraction; RMS, maximal EMG activity expressed as a percentage of maximal M-wave; VA_TMS_, maximal voluntary activation assessed by TMS; MEP, motor evoked potential; SP, silent period; MVC, measurement performed during maximal voluntary contraction; 75: measurement performed during submaximal voluntary contractions at 75% MVC; 50: measurement performed during submaximal voluntary contractions at 50% MVC; M_max_: M-wave recorded at rest*.

### MVC Force, RMS and Voluntary Activation

**Figure 4:**
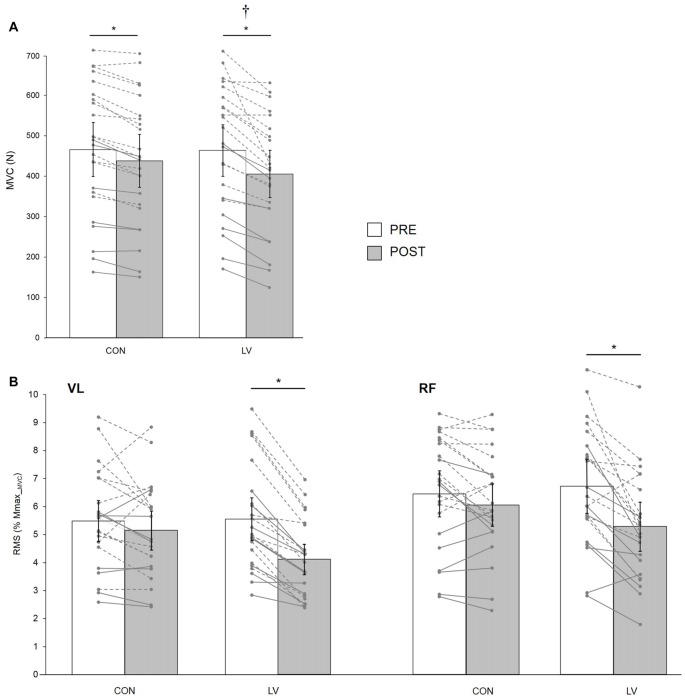
Knee extensor MVC force (MVC, **A**) and associated maximal electromyography (EMG) root-mean-square (RMS) normalized to M_max_MVC_ for VL and RF muscles **(B)** recorded before (PRE) and after (POST) a 30-min resting (CON) or LV period. The displayed data are mean values. Error bars denote the 95% confidence interval. Data from individual young (dashed lines) and old (solid lines) subjects are displayed for both testing sessions at PRE and POST measurements. *Significantly different from PRE; † significantly different from POST CON (*p* < 0.05).

There was a significant condition × time interaction for MVC (*F*_(1,21)_ = 4.95;* p* = 0.03; pη^2^ = 0.19). Although MVC was significantly decreased at POST for both CON (−6.3 ± 4.4%, *p* = 0.01) and LV groups (−12.9 ± 7.7%, *p* < 0.001), the MVC loss was significantly greater after LV (*p* = 0.001; Figure 4A). Similarly, a significant condition × time interaction (*F*_(1,21)_ = 17.42; *p* < 0.001; pη^2^ = 0.45) was found for EMG RMS measured during MVC, with a significant decrease reported POST LV for both VL (−25.1 ± 10.7%) and RF (−20.9 ± 16.5%) but no significant changes POST CON (*p* = 0.32; Figure 4B). Unlike force and RMS, there was no significant condition × time interaction for VA_TMS_ (*F*_(1,21)_ = 1.08; *p* = 0.30; pη^2^ = 0.04); however, there was a significant main effect of time (*F*_(1,21)_ = 12.15; *p* = 0.02; pη^2^ = 0.36), with decreased VA_TMS_ for POST measurements (Table 2).

**Table 2:** VA_TMS_, MEP, SP, M-wave and Pt measurements before (PRE) and after (POST) a 30-min resting (CON) or local vibration (LV) period.

	Control			Vibration		
	Pre	Post	Change in mean (95% CI)	Pre	Post	Change in mean (95% CI)
VA_TMS_ (%)	92.9 ± 6.0	91.7 ± 6.9	−1.4 (−2.7 to −0.1)	93.0 ± 6.6	90.8 ± 7.0	−2.4 (−4.1 to −0.6)
MEP_MVC_VL_ (% M_sup_100_)	46.4 ± 14.9	45.6 ± 13.8	0.5 (−8.2 to 9.1)	47.4 ± 15.2	45.7 ± 12.6	5.9 (−10.7 to 22.5)
MEP_75_VL_ (% M_sup_75_)	61.2 ± 12.5	59.5 ± 14.2	−1.4 (−6.7 to 3.7)	57.0 ± 12.2	57.8 ± 13.8	1.7 (−4.2 to 7.6)
MEP_50_VL_ (% M_sup_50_)	63.6 ± 17.3	62.1 ± 14.7	−1.2 (−9.5 to 12.0)	57.7 ± 16.7	56.0 ± 16.4	−2.3 (−7.7 to 3.1)
MEP_MVC_RF_ (% M_sup_100_)	63.0 ± 21.2	62.7 ± 18.0	2.1 (−5.4 to 9.6)	63.2 ± 18.9	64.1 ± 21.2	2.6 (−6.1 to 11.3)
MEP_75_RF_ (% M_sup_75_)	80.9 ± 19.1	77.5 ± 27.0	−5.0 (−11.0 to 1.0)	77.2 ± 15.2	81.3 ± 22.5	5.4 (−3.2 to 14.2)
MEP_50_RF_ (% M_sup_50_)	83.6 ± 20.0	81.0 ± 19.9	−2.4 (−6.3 to 1.4)	80.5 ± 13.7	83.3 ± 18.8	2.7 (−1.4 to 6.7)
MEP_BF_ (mV.s)	1.5^E−05^ ± 9.5^E−0.6^	1.4^E−05^ ± 8.2^E−0.6^	−7.8 (−24.7 to 9.1)	1.4^E−05^ ± 1.2^E−0.5^	1.2^E−05^ ± 9.2^E−0.6^	−12.0 (−32.6 to 8.4)
SP_MVC_VL_ (ms)	258 ± 75	267 ± 76	4.4 (0.1 to 8.8)	250 ± 76	262 ± 80	5.8 (0.1 to 11.6)
SP_75_VL_ (ms)	258 ± 80	273 ± 83	6.4 (2.2 to 10.7)	259 ± 79	266 ± 84	4.1 (−3.3 to 11.4)
SP_50_VL_ (ms)	262 ± 78	264 ± 76	0.8 (−2.2 to 3.9)	257 ± 73	264 ± 81	4.4 (−3.4 to 12.2)
SP_MVC_RF_ (ms)	258 ± 76	262 ± 84	1.0 (−4.5 to 6.5)	249 ± 78	263 ± 80	6.9 (1.4 to 12.3)
SP_75_RF_ (ms)	258 ± 79	265 ± 87	2.1 (−1.5 to 5.8)	247 ± 81	269 ± 84	10.0 (4.6 to 15.5)
SP_50_RF_ (ms)	255 ± 78	263 ± 85	2.3 (−1.2 to 6.0)	242 ± 79	252 ± 77	10.1 (4.0 to 16.2)
M_max_VL_ (mV.s)	0.087 ± 0.034	0.086 ± 0.031	0.5 (−3.2 to 4.3)	0.088 ± 0.029	0.087 ± 0.029	−0.5 (−3.2 to 2.1)
M_max_RF_ (mV.s)	0.063 ± 0.019	0.063 ± 0.018	0.5 (−2.3 to 3.4)	0.060 ± 0.015	0.059 ± 0.015	−0.9 (−3.5 to 1.7)
M_sup50_VL_ (mV.s)	0.069 ± 0.028	0.066 ± 0.025	−2.2 (−6.7 to 2.2)	0.070 ± 0.026	0.068 ± 0.024	−2.3 (−5.2 to 0.4)
M_sup50_RF_ (mV.s)	0.055 ± 0.018	0.046 ± 0.014	−15.6 (−19.2 to 12.0)	0.051 ± 0.013	0.042 ± 0.011	−17.2 (−21.7 to 12.8)
Pt (N)	132 ± 43	117 ± 37	−10.7 (−13.7 to 7.7)	137 ± 50	121 ± 47	−11.3 (−15.6 to 7.0)

*VA_TMS_, maximal voluntary activation assessed by TMS; MEP, motor evoked potential; SP, silent period; MVC, measurement performed during maximal voluntary contraction; 75: measurement performed during submaximal voluntary contractions at 75% MVC; 50: measurement performed during submaximal voluntary contractions at 50% MVC; VL, vastus lateralis; RF, rectus femoris; BF, biceps femoris; M_max_, M-wave recorded at rest; 95% CI, 95% confidence interval*.

### MEP, SP, M-wave and Pt

Condition × time × muscle interactions were not significant for MEP, SP or M-wave at any contraction strength (*F*_(1,21)_ = 0.11–1.74; *p* > 0.05; pη^2^ = 0.04–0.21; Table 2). However, in some instances, there was a main effect of time (Table 1) such that SP duration increased and M-wave area decreased from pre- to post-intervention for pooled CON and LV data (*p* < 0.05, Table 2). The condition × time interaction was not significant for Pt (*F*_(1,21)_ = 0.02; *p* = 0.88; pη^2^ = 0.01); however, there was a significant main effect of time (*F*_(1,21)_ = 46.64; *p* < 0.001; pη^2^ = 0.71), with decreased Pt for POST measurements (Table 2).

### TMEP and MEP/TMEP Ratio

**Figure 5:**
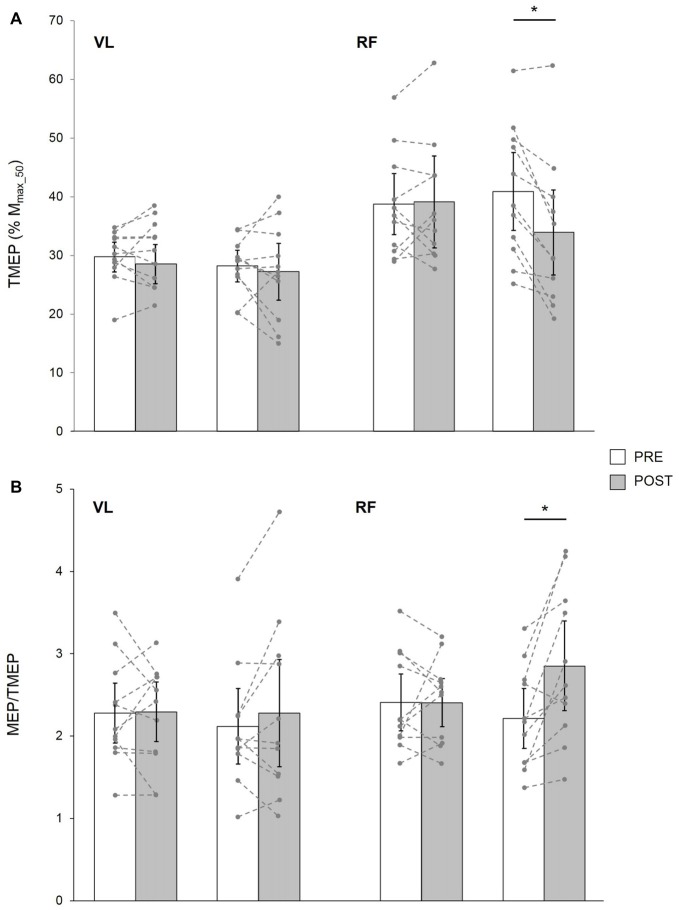
TMEP normalized to M_max_50_ **(A)** and MEP/TMEP ratios **(B)** recorded in 11 healthy young subjects for VL and RF muscles before (PRE) and after (POST) a 30-min resting (CON) or LV period. The displayed data are mean values. Error bars denote the 95% confidence interval. Data from the individual subjects are displayed (dashed lines) for both testing sessions at PRE and POST measurements. *Significantly different from PRE (*p* < 0.05).

Based on data from 11 young subjects, a significant condition × time × muscle interaction was found for TMEP (*F*_(1,10)_ = 16.95; *p* = 0.01; pη^2^ = 0.32). It was significantly decreased POST LV for the RF (*p* = 0.001) but not the VL (*p* = 0.27), while it remained unchanged after CON for both muscles (*p* = 0.21 and *p* = 0.67 for VL and RF, respectively; Figure 5A).

In these subjects, a significant condition × time × muscle interaction was found for MEP/TMEP ratio (*F*_(1,10)_ = 9.83; *p* = 0.01; pη^2^ = 0.39). It was significantly increased POST LV on the RF (*p* = 0.002) while it was unchanged for VL (*p* = 0.15). No significant changes were detected after CON for either RF (*p* = 0.96) or VL (*p* = 0.85) muscles (Figure 5B).

## Discussion

The principal aim of this study was to investigate the effects of a single bout of prolonged LV on knee extensor force production capacities and associated corticospinal properties in healthy adults. In the current study, LV was applied for a prolonged (30-min) period. While it has been reported that the efficiency of the spinal loop is increased during the first seconds of exposure to the LV stimulus and may thus in turn evoke an extra voluntary force (Magalhães and Kohn, 2010; Magalhães et al., 2013), prolonged exposure has been reported to reduce spinal excitability (Fry and Folland, 2014) and maximal muscle output (Jackson and Turner, 2003; Saito et al., 2016a), with associated potential supraspinal adaptations (Smith and Brouwer, 2005; Lapole et al., 2012b). However, we recently showed that repeated sessions of prolonged LV can enhance both neural drive and maximal strength (Souron et al., 2017). Here, we report a reduction in knee extensor MVC torque after a single bout of prolonged LV, which is accompanied by: (i) a reduction of maximal EMG activity for both VL and RF muscles, as well as, in young adults; (ii) a significant decrease in TMEP areas; and (iii) increase in MEP/TMEP ratio for RF only.

This study reported a significant MVC decrease after a 30-min prolonged LV period, which is consistent with previous results obtained for knee extensor (Kouzaki et al., 2000; Konishi et al., 2002, 2009; Jackson and Turner, 2003; Richardson et al., 2006; Saito et al., 2016a), plantar flexor (Yoshitake et al., 2004; Ushiyama et al., 2005; Herda et al., 2009) and first dorsal interosseous muscles (Shinohara et al., 2005) following a 20–30 min LV period. For the present study, there was an unexpected decrease of MVC force for CON, i.e., after 30-min of rest. This may be explained by fatigue induced by the large number of contractions that subjects had to perform, as supported by the significant reduced of Pt for CON. Yet, the greater force loss observed for LV confirms the potential for LV to decrease force generation capacity.

To our knowledge, this was only the second study to compare the after-effects of prolonged LV between young and old subjects. The decrease in MVC was similar for young (−11.9 ± 8.4%) and old (−14.3 ± 7.2%) subjects, which contradicts the previous report of a greater MVC decrease in young than old subjects (Richardson et al., 2006). These authors suggested a gamma loop function impairment in the old due to an age-related decrease in number and size of type II muscle fibers (Snyder-Mackler et al., 1994). Indeed, the decrease in Ia afferent inputs onto alpha motoneurons has been suggested to predominantly affect the recruitment of high-threshold motoneurons that supply fast-twitch muscle fibers (Bongiovanni et al., 1990). Regardless of the mechanisms, the discrepancy between our results and those reported by Richardson et al. (2006) indicates a need for further investigation of LV-related effects in older adults. This is particularly important given the limited sample size in our study (*n* = 8) and the one conducted by Richardson et al. (2006, *n* = 7).

The ability to produce a maximal level of voluntary force depends on both peripheral and central mechanisms. Evidence based on electrically-evoked twitch (Ushiyama et al., 2005; Herda et al., 2009; Fry and Folland, 2014; Saito et al., 2016a,b) and M-wave amplitudes (Ushiyama et al., 2005; Ekblom and Thorstensson, 2011; Fry and Folland, 2014; Cattagni et al., 2016; Farabet et al., 2016; Saito et al., 2016a,b) seems to rule out an influence of peripheral mechanisms on the MVC decrease commonly seen following prolonged LV. Our results further confirm this interpretation, as there were no differences between CON and LV for: (i) M-wave areas evoked on either relaxed or contracted muscles; and (ii) potentiated peak twitch. Hence, an alteration to the neural command probably fully explains the decrement in force-generation capacity after prolonged LV. In the present study, although cortical voluntary activation (VA_TMS_) was decreased minutely over time, and so indicating some central fatigue in line with MVC impairment in both conditions, it was independent of the condition, i.e., no condition × time interaction effect, which is in agreement with what we recently reported for dorsiflexor muscles (Farabet et al., 2016). The LV-induced reduction in maximal EMG activity for both VL and RF muscles corroborates previous findings for knee extensor (Kouzaki et al., 2000; Konishi et al., 2002, 2009; Jackson and Turner, 2003; Richardson et al., 2006), plantar flexor (Yoshitake et al., 2004; Ushiyama et al., 2005; Herda et al., 2009) and first dorsal interosseous muscles (Shinohara et al., 2005). Based on EMG results, and because vibration-induced excitatory Ia afferents project to both the spinal cord (Eklund and Hagbarth, 1965; De Gail et al., 1966) and cortical structures (Goodwin et al., 1972; Roll and Vedel, 1982; Roll et al., 2012), the decreased MVC observed after LV exposure may be related to a neural drive alteration occurring at a spinal and/or supraspinal level. It is thus likely that VA_TMS_ was not sensitive enough to detect such neural modulations after an acute exposure to LV.

Although not investigated in the present study, previous results show H-reflex depression after prolonged LV (Heckman et al., 1984; Ushiyama et al., 2005; Ekblom and Thorstensson, 2011; Lapole et al., 2012a,b; Fry and Folland, 2014; Farabet et al., 2016), suggesting an impaired motoneuron excitability with LV. As a measure of motoneuron excitability, the H-reflex suffers the disadvantage to be potentially influenced by presynaptic inhibition at the Ia afferents level (Hultborn et al., 1996). Hence, one of the most novel aspects of this study was to assess motoneuronal excitability in healthy young adults via stimulation of the corticospinal tract (TMEP) in order to avoid an influence of classical presynaptic inhibition (Nielsen and Petersen, 1994; Jackson et al., 2006). In the present study, TMEP_RF_ was reported to be significantly decreased following the 30-min LV period, while it remained unchanged for TMEP_VL_. This discrepancy may be explained by the position of the vibratory device on the leg (i.e., over the RF muscle). This likely led to a stronger activation of Ia afferents supplying the RF muscle rather than the VL. The observed reduction in TMEP_RF_ after prolonged LV may imply: (i) impaired excitability of the corticospinal descending tracts; (ii) impaired efficacy in synaptic transmission between corticospinal descending tracts and alpha motoneurons; and/or (iii) impaired intrinsic motoneuronal properties (Giesebrecht et al., 2010). Although different from a vibration intervention, studies of muscle fatigue failed to demonstrate impaired excitability of the corticospinal tract (Petersen et al., 2003; Giesebrecht et al., 2010) and impaired efficacy in synaptic transmission during strong contractions (Petersen et al., 2003). Hence, we believe that a reduction in intrinsic motoneuronal excitability is the most likely explanation for our observed decrease in TMEP size following prolonged LV. Such acute modulations in motoneuronal properties may be a plausible explanation to the functional effects reported after longer-term use (Lapole and Pérot, 2010; Iodice et al., 2011; Lapole et al., 2013; Tankisheva et al., 2015; Souron et al., 2017). However, these results should be interpreted with caution since TMEP data were collected from only 11 young subjects, reducing the statistical power for this analysis. Also, it remains to be investigated whether or not such adaptations could occur in old subjects.

It is worth noting that MEPs, either evoked during maximal or submaximal voluntary contractions, were unchanged following the 30-min prolonged LV period on RF and VL muscles. This is consistent with some (Rosenkranz and Rothwell, 2004, 2006; Smith and Brouwer, 2005; Lapole et al., 2012b; Lapole and Tindel, 2015) but not all studies. Indeed, previous literature has reported either increased (Steyvers et al., 2003; Smith and Brouwer, 2005; Christova et al., 2011; Lapole et al., 2012b) or decreased (Steyvers et al., 2003; Farabet et al., 2016) MEPs after prolonged LV using vibration frequencies and amplitudes from 25 Hz to 100 Hz and 0.2–2 mm, respectively (i.e., comparable to the 100 Hz and 1 mm of the present study). Differences in the investigated muscles and the vibration parameters may account for these discrepancies. Also, the method used to determine the optimal TMS intensity (i.e., stimulus-response curve vs. active or resting motor threshold) may contribute to the differences reported in MEP behavior between these studies and ours. Importantly, positioning our MEP data in the literature is difficult because none of the aforementioned studies included a measure of motoneuron excitability so do not include speculation on cortical and spinal influences on the MEP. Although MEP size did not change after LV, the reduction in the TMEP_RF_ (observed for 11 young subjects) suggests that cortical excitability increased with LV in order to compensate for the reduced excitability at the spinal level; i.e., without an increase in cortical excitability, the MEP would also be reduced by the lower motoneuronal excitability. This presumed increase in cortical excitability is shown by the larger MEP/TMEP ratio after LV. However, this ratio should be interpreted with caution because the size of the MEP and TMEP were not matched and the potentials were separated by ~1–4 min. Indeed, because TMS intensity was set to investigate VA, the MEP is approximately twice the size of the TMEP, so is likely testing a far greater proportion of the motoneuron pool than the TMEP.

Although SP duration increased in some conditions (at MVC and 75% MVC for VL and RF, respectively), this was independent of the condition since no interaction effect was found. Thus, similar to our recent findings in the dorsiflexors (Farabet et al., 2016), it appears that LV had no impact on SP duration. This suggests that acute prolonged LV did not alter GABA_B_ neurotransmission (Werhahn et al., 1999) of inhibitory neurons within the primary motor cortex projecting onto the pyramidal cells (Inghilleri et al., 1993). The technique of paired-pulse TMS should be considered in parallel to SP measurement for future studies since it has been reported as the more appropriate method to measure changes in intracortical inhibition (Kidgell and Pearce, 2010).

In a small sample of young adults (*n* = 6), it has been reported that the effect of brief (20 s) periods of high-frequency vibration on neuronal pathways is strongly time-dependent (Magalhães et al., 2013). Prolonged LV is unlikely to induce effects with such a strong dependence on time, but it is probable that the relative importance of different mechanisms would vary immediately after, shortly after or at longer periods after stimulation. Hence, it would be important for future work to assess the variables of the current study at a broader range of times following prolonged LV.

In conclusion, the significant decrease of MVC reported in this study after 30-min of LV in an adult population seems to be explained primarily by a reduced output from the motoneuron pool, as demonstrated by the reduction in maximal EMG activity for VL and RF in both young and old subjects. Moreover, at least for young subjects, a reduced motoneuron excitability may be involved as indicated by decreased TMEP area for RF, this finding being reported for the first time in this scientific framework. The current results for young subjects also show a concomitant increase in cortical excitability after prolonged LV as indicated by the increased MEP/TMEP ratio, likely occurring to compensate for reduced excitability at the motoneurons. Although the absence of TMEP data from old subjects prevents speculation on motoneuron excitability in this group, similar findings of reduced force generation capacities and EMG activity for both young and old subjects may suggest that LV could be as efficient to induce neural adaptations in old subjects. In support of this suggestion, positive long-term adaptations to prolonged LV (e.g., increased maximal strength) have been shown in both young (Lapole and Pérot, 2010; Lapole et al., 2013; Souron et al., 2017) and old adults (Tankisheva et al., 2015). Notably, any speculation on the effects of LV in the elderly is limited by our small number of older adults, so future research to elucidate the mechanisms of prolonged LV must employ a larger sample size.

Although the functional link between the recorded parameters and motor performance remains to be elucidated, our findings suggest that a single bout of prolonged LV has the potential to induce neural modulations in healthy adults. Current and previous findings (e.g., Tankisheva et al., 2015; Souron et al., 2017) suggest it is conceivable that LV could trigger chronic neural adaptations in both healthy young and old subjects that may eventually lead to an improvement in muscle performance. If, in the future, this proposition is supported by stronger experimental findings (especially with a larger sample size for the old population), LV could be considered as an efficient and alternative training method to the ones classically used (e.g., resistance training, neuromuscular electrical stimulation), but which have identified limitations in rehabilitation settings. For example, resistance training may not be applicable to everyone, especially those with significant motor impairments that limit the capacity for volitional exercise (e.g., old or clinical populations). Neuromuscular electrical stimulation avoids such an issue and has been shown to acutely modulate the central nervous system (Bochkezanian et al., 2017) and to improve muscle performance in clinical (Hauger et al., 2017) or old (de Oliveira Melo et al., 2016) populations. However, some limitations (e.g., pain, restricted spatial recruitment of muscle fibers) are commonly associated with this technique (Maffiuletti, 2010) that may make LV a more attractive method for some sensitive subjects.

## Author Contributions

RS was involved in the conception of the research, the acqusition, analysis and interpretation of the data. RS was also involved in the revision of the manuscript. TB was involved in the conception of the research, the acquisition, analysis and interpretation of the data, as well as in the revision of the manuscript. GYM, TL and CJM were involved in the conception of the research design, the analysis and interpretation of the results and the writing of the manuscript (revising the work). As the contributing author, GYM gave his final approval for submission.

## Conflict of Interest Statement

The authors declare that the research was conducted in the absence of any commercial or financial relationships that could be construed as a potential conflict of interest.

## Abbreviations

BF: Biceps femoris
EMG: Electromyography
ERT: Estimated resting twitch amplitude
LV: Local vibration
M_max_: Maximal M-wave recorded on relaxed muscles
MEP: Motor evoked potential
MVC: Maximal voluntary contraction
PNS: Peripheral nerve stimulation
RF: Rectus femoris
RMS: Root mean square
SIT: Superimposed twitch amplitude
SP: Silent period
Pt: Potentiated peak twitch
TMEP: Thoracic motor evoked potential
TMS: Transcranial magnetic stimulation
VA_TMS_: Voluntary activation assessed with TMS
VL: Vastus lateralis.
